# Utility of ^68^Ga-DOTATATE PET-MRI for Gamma Knife^®^ stereotactic radiosurgery treatment planning for meningioma

**DOI:** 10.1093/bjr/tqad026

**Published:** 2023-12-12

**Authors:** Gishan Ratnayake, Michael Huo, Akash Mehta, Prabhakar Ramachandran, Mark B Pinkham, Phillip Law, Trevor Watkins, Sarah Olson, Bruce Hall, Simon Brown, Ryan Lusk, Catherine Jones, Eoin O’Mahoney, George McGill, Matthew C Foote

**Affiliations:** Department of Radiation Oncology, Princess Alexandra Hospital, Brisbane 4102, Australia; Faculty of Medicine, University of Queensland, Brisbane 4006, Australia; Department of Radiation Oncology, Princess Alexandra Hospital, Brisbane 4102, Australia; Faculty of Medicine, University of Queensland, Brisbane 4006, Australia; Department of Radiation Oncology, Princess Alexandra Hospital, Brisbane 4102, Australia; Faculty of Medicine, University of Queensland, Brisbane 4006, Australia; Department of Radiation Oncology, Princess Alexandra Hospital, Brisbane 4102, Australia; Faculty of Medicine, University of Queensland, Brisbane 4006, Australia; Department of Radiation Oncology, Princess Alexandra Hospital, Brisbane 4102, Australia; Faculty of Medicine, University of Queensland, Brisbane 4006, Australia; Faculty of Medicine, University of Queensland, Brisbane 4006, Australia; Department of Medical Imaging, Princess Alexandra Hospital, Brisbane 4102, Australia; Faculty of Medicine, University of Queensland, Brisbane 4006, Australia; Department of Medical Imaging, Princess Alexandra Hospital, Brisbane 4102, Australia; Department of Medical Imaging, Princess Alexandra Hospital, Brisbane 4102, Australia; Department of Neurosurgery, Princess Alexandra Hospital, Brisbane 4102, Australia; Department of Medical Imaging, Princess Alexandra Hospital, Brisbane 4102, Australia; Department of Neurosurgery, Princess Alexandra Hospital, Brisbane 4102, Australia; Department of Medical Imaging, Princess Alexandra Hospital, Brisbane 4102, Australia; Department of Neurosurgery, Princess Alexandra Hospital, Brisbane 4102, Australia; Department of Radiation Oncology, Princess Alexandra Hospital, Brisbane 4102, Australia; Department of Radiation Oncology, Princess Alexandra Hospital, Brisbane 4102, Australia; Department of Medical Imaging, Princess Alexandra Hospital, Brisbane 4102, Australia; Department of Radiation Oncology, Princess Alexandra Hospital, Brisbane 4102, Australia; Department of Radiation Oncology, Princess Alexandra Hospital, Brisbane 4102, Australia; Faculty of Medicine, University of Queensland, Brisbane 4006, Australia

**Keywords:** MRI, PET, DOTATATE, GK, SRS, meningioma

## Abstract

**Objectives:**

To investigate the impact of adding ^68^Ga-DOTATATE PET/MRI to standard MRI for target volume delineation in Gamma Knife^®^ stereotactic radiosurgery (GKSRS) for meningioma.

**Methods:**

Seventeen patients with 18 lesions undergoing GKSRS for WHO grade 1 meningioma were enrolled in a prospective study. All patients underwent pre-treatment ^68^Ga-DOTATATE PET/MRI examination in addition to standard procedures. Five clinicians independently contoured the gross tumour volume (GTV) based on standard MRI (GTV_MRI_) and PET/MRI (GTV_PET/MRI_) on separate occasions. Interobserver agreement was evaluated using Cohen’s Kappa statistic (CKS), Dice similarity coefficient (DC), and Hausdorff distance (HD). Statistical analysis was performed with paired *t*-test and Wilcoxon signed rank test.

**Results:**

The addition of PET/MRI significantly increased GTV contour volume (mean GTV_PET/MRI_ 3.59 cm^3^ versus mean GTV_MRI_ 3.18 cm^3^, *P* = .008). Using the treating clinician’s pre-treatment GTV_MRI_ as the reference, median CKS (87.2 vs 77.5, *P* = .006) and DC (87.2 vs 77.4, *P* = .006) were significantly lower, and median HD (25.2 vs 31.0, *P* = .001) was significantly higher with the addition of PET/MRI. No significant difference was observed in interobserver contouring reproducibility between GTV_MRI_ and GTV_PET/MRI_.

**Conclusion:**

The addition of ^68^Ga-DOTATATE PET/MRI for target volume delineation in GKSRS for meningioma is associated with an increase in GTV volume and greater interobserver variation. PET/MRI did not affect interobserver contouring reproducibility.

**Advances in Knowledge:**

This study provides novel insights into the impact of ^68^Ga-DOTATATE PET/MRI on GTV delineation and interobserver agreement in meningioma GKSRS, highlighting its potential for improving GKSRS treatment accuracy.

## Introduction

Meningiomas are mostly benign tumours and represent about 30% of all primary intracranial tumours.^1^ Gamma Knife^®^ Stereotactic Radiosurgery (GKSRS) is a non-invasive alternative to surgery for select tumours offering comparable local control outcomes.^2^ GKSRS for meningioma involves the precise delivery of 12-15 Gy of ionizing radiation in a single session whilst minimising dose to surrounding normal tissue.^3^ Local control rates for WHO grade 1 meningiomas exceed 90% at 5 years after GKSRS.^4^

Given the steep dose gradients expected with GKSRS, accurate target definition is critical, particularly for tumours in close proximity to critical structures including the optic pathways, brainstem, and pituitary.^5^MRI with gadolinium contrast is the current standard for tumour delineation. However, the base of skull has complex anatomy with multiple contrast-enhancing structures including the cavernous sinus, arteries, and pituitary gland. This, along with MRI artefacts, bone infiltration and the presence of scar tissue after surgery can represent significant challenges to accurately defining tumour extent.^6^ MRI also provides little information on what extent of dural tail could harbour disease that should be included within the treatment volume.^7^

The introduction of molecular imaging with PET/MRI has the potential to improve target volume delineation for GSKRS. DOTA-_D_Phe, ^1^Tyr^3^-octreotate (DOTATATE) is a radiotracer that binds to the somatostatin receptor (SSTR2), which is highly expressed in meningioma.^8^ Early studies have demonstrated the feasibility of an alternative octreotide analogue ^68^Ga-DOTATOC imaged with PET and CT, which led to significant modification of treatment volumes in meningioma.^9–12^ However, the use of simultaneous PET/MRI is still under investigation when compared to modern high definition MRI planning.

The objective of this study was to assess the value of ^68^Ga-DOTATATE PET/MRI in comparison to MRI alone for GKSRS treatment planning for meningioma. We assessed both the definition of the gross tumour volume (GTV) size and the agreement in contours among clinicians.

## Materials and methods

### Imaging

Patients due to receive GSKRS for meningioma were prospectively enrolled into an institutional ethics approved study (HREC/16/QPAH/043). All patients had standard GKSRS imaging procedures which include an MRI (T1 weighted with gadolinium contrast and T2 thin sequences) on the day of radiosurgery, and a planning CT scan. Both were done with a Leksell stereotactic head frame in place, with the MRI co-registered to the CT. In addition, participants underwent a ^68^Ga-DOTATATE PET/MRI examination using a commercially available system (Siemens Biograph mMR) prior to the day of radiosurgery. A 30 min PET acquisition of the brain was performed 45 min after a single intravenous bolus injection of 60 MBq of ^68^Ga-labelled DOTATATE in a total volume of 10 mL or less. MRI acquisition was performed synchronously with the PET. This was subsequently fused with the day-of-treatment dataset, and reviewed post-treatment for the generation of study contours.

### Target volume delineation

The PET/MRI data were loaded into the GammaPlan^®^ software (version 11.1.1) and co-registered for each participant. It is noted that, at the time of the study, the GammaPlan software was unable to recognize the standardized uptake value (SUV) data or the window levels (0-5 SUV) saved in the DICOM headers of the PET datasets. For this reason, the pixel values corresponding to 0 and 5 SUV for the 16 bit PET images were calculated manually using dose data from the DICOM headers, entered manually into the GammaPlan software (for further details please refer to the Supplementary Material). Five clinicians (including specialists in radiation oncology and neurosurgery trained in GKRS) independently contoured the gross tumour volume (GTV), defined as the entire meningioma visible for treatment. All contours were completed in the same sitting. The GTV_MRI_ was contoured on MRI alone. Once completed, the PET was opened in an additional screen side-by-side with the MRI still visible, and the GTV_PET/MRI_ contours were generated.

GTV defined on the MRI and CT alone was designated GTV_MRI_ and after incorporating the PET/MRI GTV_PET/MRI_. Clinicians were blinded to the PET/MRI images until after GTV_MRI_ had been defined and PET/MRI was not used in the actual treatment of the patients.

### Contour analysis

To assess interobserver agreement, we used three metrics to evaluate the comparison between a reference contour and the additional clinicians’ contours for each case defined on MRI and PET/MRI; Cohen’s Kappa statistic (CKS), Dice similarity coefficient (DC), and Hausdorff distance (HD). CKS is a measure of agreement between contours and is defined as the difference between the actual agreement (called “observed” agreement) compared to the expected agreement to be present by chance alone (called “expected” agreement). It outputs a value ranging between −100 and 100; complete disagreement (−100), no agreement above chance (0), and a perfect match (100).^13^ DC calculates the similarity of two entities by calculating the common pixels of the reference contour and other contours and compares them against the addition of total pixel values of the two contours.^14^ The coefficient ranges between 0 and 100, indicating the extent of overlap. HD measures the maximum distance (in mm) between a contour and the reference contour and vice-versa. The magnitude of the HD establishes a correlation between two contours, for example, a smaller HD implies a higher degree of similarity.^15^

**Figure 1.**
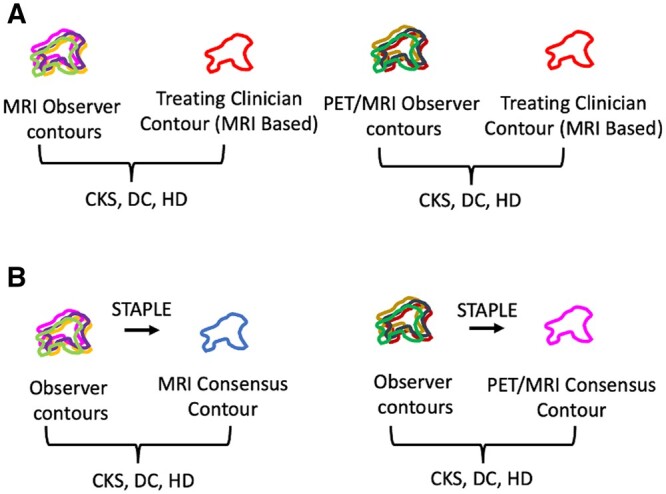
Contour analysis workflow for (A) comparison to MRI-based treating clinician reference contour and (B) interobserver variability assessment using a simultaneous truth and performance level estimation contour. Abbreviations: CKS = Cohen Kappa Score; DC: Dice Coefficient; HD: Hausdorff Distance.

We assessed the interobserver agreement between the MRI and PET/MRI contours when compared to the reference GTV_MRI_ to evaluate the effect of adding PET/MRI to target volume delineation across the five clinicians (Figure 1A). We also compared the GTV size between the two contour datasets. To assess the effect on interobserver variability, we created a separate consensus contour using the simultaneous truth and performance level estimation (STAPLE) algorithm for both the MRI (STAPLE_MRI_) and PET/MRI data (STAPLE_PET/MRI_). The STAPLE computes a probabilistic estimate of the “ground truth” contour that represents the desired tumour volume.^16^^,^^17^ We then compared the GTV_MRI_ contours to the STAPLE_MRI_ contour and similarly the GTV_PET/MRI_ contours to the STAPLE_PET/MRI_ contour (Figure 1B). Two radiation oncologists performed a qualitative review of the volumes on a case-by-case basis, with all clinician contours visible. Areas of inconsistency and adjacent PET-avid structures were determined by direct visualization and agreement between the two clinicians.

### Statistical analysis

The mean volumes of the MRI contour and the PET/MRI were compared for each observer using a paired *t*-test. A paired analysis of the CKS, DCE, and HD was completed with a Wilcoxon signed rank test. Statistical analysis was completed with GraphPad Prism (version 9.4.0). Statistical significance for all tests was deemed as *P* < .05.

## Results

From September 2016 to February 2020, 17 participants with 18 meningiomas (either radiologically-diagnosed, or histologically confirmed to be grade 1; *n* = 4) for GKSRS were recruited. Median age was 54 years and the majority were female (*n* = 14). The meningiomas were located mainly in the cerebellopontine angle (*n* = 6) and cavernous sinus (*n* = 5). Additional patient characteristics and contour comparisons can be found in Table 1. No patients had a history of irradiation.

**Table 1.** Patient characteristics and mean GTV contour comparison metrics.

Patient	Tumour location	Tumour volume (cm^3^)	Variation regions	Previous surgery
1	Sphenoid ridge	1.90	Vessel, bone, optic nerve	No
2	Cerebellar tentorium	2.47	Tentorium, dural tail	No
3	Cavernous sinus	2.07	Tentorium	No
4	Sphenoid ridge	5.55	ICA, cavernous sinus	No
5	Cavernous sinus	4.78	Pituitary ICA, petrous apex	No
6	CPA	1.78	Vessels, tentorium	No
7	Cerebellar tentorium	4.44	Tentorium	Yes
8	CPA	3.96	Jugular vessel, dura	No
9	Cavernous sinus	5.01	Tentorium, Meckel's cave, ICA, dura	No
10	CPA	3.04	Tentorium	No
11	CPA	3.18	IAM	No
12	Cerebellar tentorium	4.74	Tentorium	Yes
13	Cavernous sinus	4.29	Pituitary, ICA, Meckel’s	No
14	Frontal	6.63	Ethmoid	No
15	CPA	6.15	Tentorium, clivus	Yes
16	Cavernous sinus	0.84	Cavernous sinus	No
17	CPA	1.12	Tentorium	No
18	Frontal	6.53	Tumour/bone interface	Yes

From the 18 meningiomas studied and contoured by the five clinicians, 89 pairs of contours (GTV_MRI_ and GTV_PET/MRI_) were included in the analyses. One pair of contours was discontinuous on an axial slice preventing a STAPLE volume calculation, and hence was excluded from the analysis using STAPLE as a reference contour. Summary of DOTATE PET/MRI parameters are available in Supplementary Table S1.

**Figure 2.**
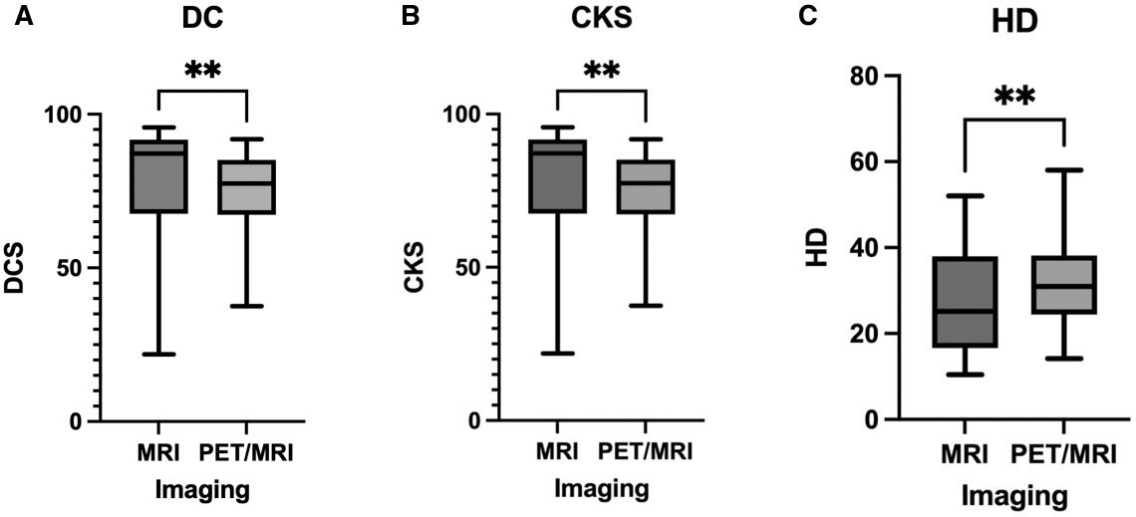
Comparison of (A) Dice Coefficient, (B) Cohen’s Kappa Score, and (C) Hausdorff Distance (HD) between MRI versus PET/MRI contours in comparison to the MRI-based reference contour. **P < .05.

**Figure 3.**
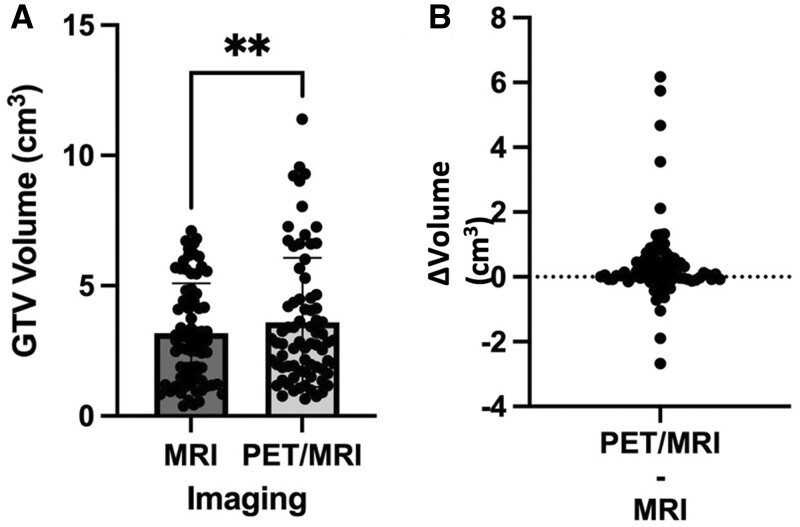
(A) Comparison of GTV volume (cm^3^) by imaging modality and (B) difference plot. **P < .05.

There was greater interobserver disagreement with the addition of PET to MRI when compared to the MRI reference contour alone (Figure 2). The median CKS (87.2 vs 77.5, *P* = .006) and DC (87.2 vs 77.4, *P* = .006) were significantly lower and the HD (25.2 vs 31.0, *P* = 0.001) was significantly higher. On a paired analysis (Figure 3A), the mean GTV_PET/MRI_ contour volume (3.59 cm^3^ ± 2.48 standard deviation, SD) was significantly larger than the mean GTV_MRI_ contour volume (3.18 cm^3^ ± 1.91, *P* = .008). Out of 18 lesions, the GTV_PET/MRI_ contour volume was larger in 14 lesions. Some of the volumes had large changes in contour with use of GTV_PET/MRI_, with the volume size increasing more than 2 cm^3^ in five cases, with the largest change being 6.2 cm^3^ (Figure 3B).

**Figure 4.**
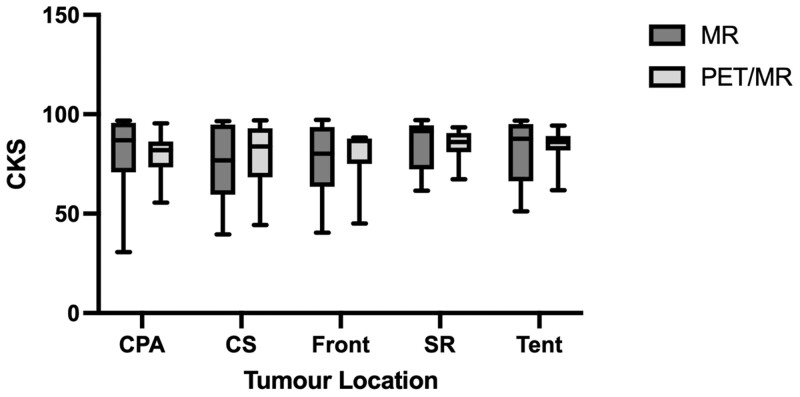
Comparison of Cohen’s Kappa Score by tumour site with simultaneous truth and performance level estimation contours. Abbreviations: CPA = cerebellopontine angle; CS = cavernous sinus; Front = frontal; SR = sphenoid ridge; Tent = cerebellar tentorium. *P* > .05 for all comparisons.

There was no significant change in interobserver contouring reproducibility with the use of PET/MRI when the volumes were compared to their respective STAPLE volumes. The median CKS was similar with GTV_MRI_ compared to GTV_PET/MRI_ (83.8 vs 86.6, *P* = .82), as was the DC (86.2 vs 85.6, *P* = 0.11) and the HD (27.9 vs 28.8, *P* = 0.63). The CKS was also noted to be similar in all tumour subsites regardless of the imaging modality (Figure 4).

**Figure 5.**
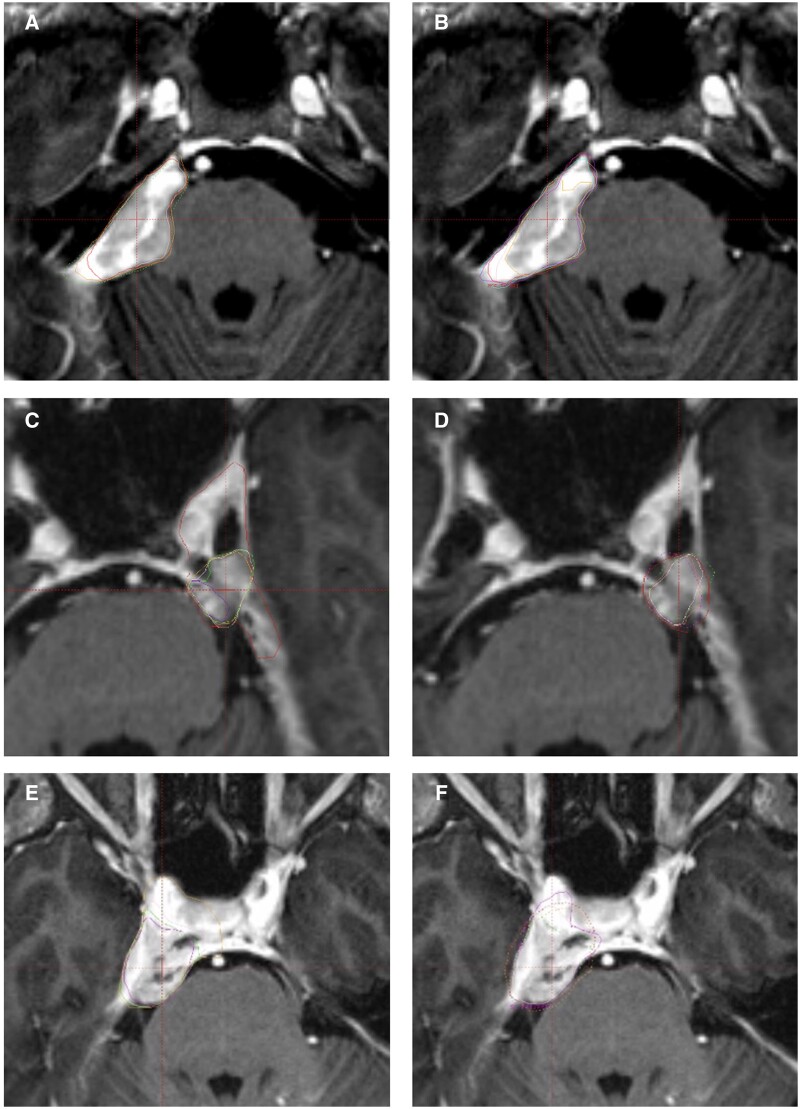
Cerebellopontine angle tumour GTV volumes comparing MRI (A) with PET/MRI (B). Cavernous sinus tumour volumes comparing MRI (C, E) and PET/MR (D, F).

On qualitative review, there were four lesions where the PET/MRI resulted in a smaller volume and these were located in the sphenoid ridge, tentorium, cerebellopontine angle (CPA), and Meckel’s cave. There were 14 lesions which were larger on PET/MRI; four located in the CPA and four in the cavernous sinus. For CPA tumours, the most common area of inter-observer variation was along the tentorium, for which the PET/MRI subjectively improved consistency (Figure 5A and B). For cavernous sinus lesions, the greatest area of uncertainty was extension along the tentorium and the degree of encasement of the carotid arteries, which the PET/MRI subjectively improved (Figure 5C-H).

## Discussion

We report one of the first studies investigating the use of ^68^Ga-DOTATATE PET/MRI for treatment of patients with intact meningioma with GKRS. Our study demonstrated a significant change in GTV contouring when incorporating PET/MRI data in comparison to MRI alone for all five clinicians but overall no significant changes in interobserver agreement across all metrics.

The addition of PET/MRI in our study led to an increase in target volume size, with a small number substantially larger. This is similar to the findings of an early study by Gehler et al. using [68]-Ga-DOTATOC PET/CT which found that the PET-based GTV was larger in 50% of patients compared to the MRI.^12^ However, other studies such as Graf et al. which found the addition of [68]-Ga-DOTATOC PET/CT data led to an overall reduction in the GTV volume.^9^ They note, however, the addition of PET led to on average 1.5 cm^3^ additional volume being included which was otherwise missed with MRI alone. A recently published study that investigated the addition [68]-Ga-DOTATATE PET/MR for both post-operative (18/25) and intact (7/25) meningiomas reported that the overall median volume was lower with PET/MRI.^18^ However, when evaluating intact meningiomas, only there was an increase in the median GTV from 14.96 cm^3^ (IQR 2.96-34.27) to 16.67 cm^3^ (IQR 3.30-55.18) with addition of PET/MRI. Volume change alone is not enough to assess the impact of novel diagnostic tools such as PET/MRI, as clinically significant changes could result in either inclusion of otherwise-equivocal but PET-avid tissue, or exclusion of non-PET avid contoured tissue.

Intuitively, PET should be able to improve contouring consistency, but we found the inter-observer agreement was no better with the addition of PET/MRI. It is possible that the benefit of PET/MRI is predominantly for tumours in particular locations, and thus this effect was diluted within the entire cohort. On qualitative review, this has appeared to be the case for CPA, tentorium, and cavernous sinus lesions; however, further studies with greater lesion numbers are needed to verify this finding. However, this was not reflected quantitatively, and may be due to inconsistent effects across all locations. For example, for a meningioma in the cavernous sinus, PET may be helpful to define GTV in some directions but less so towards the pituitary and carotid arteries, which are also intrinsically PET-avid normal structures. A study by Maclean et al. also noted substantial interobserver variability with contouring, which only slightly improved with PET/CT and was not further improved with PET/MRI.^19^ A challenge is the lack of SUV threshold, leading to a variation in practice in areas of mildly increased avidity and a greater reliance on the MRI to contour the margins of the tumour.^20^ PET resolution is also less than MRI, potentially reducing its application in regions of irregularly-shaped targets.

Several studies describing the utility of 68-Ga DOTATATE PET/MRI in the post-treatment setting for meningiomas, following surgery or radiation therapy. A series of 20 patients with WHO Grade 2-3 lesions demonstrated that it assisted with differentiating reactive dural thickening from treatment from progressive tumours and also found additional meningiomas not seen on MRI.^21^ 68-Ga DOTATATE has shown particular benefit in delineating bone involvement.^22^ Another series of 37 cases of meningioma after a gross total resection found that PET/MRI was able to predict the presence of residual disease with an accuracy of 90%.^23^

Strengths of this study included prospective recruitment, the blinding of observers to each other, and to the PET/MRI images prior to GTV delineation, and the use of the consensus STAPLE method to analyse the target volumes. Limitations for this study were the relatively small number of observers, limited clinical information provided at the time of GTV delineation, and no consensus agreement on how to best apply the PET/MRI data. Additionally, there were small numbers of lesions within each anatomical subsite, limiting analyses of PET/MRI utility based on location.

In summary, for meningioma GKSRS planning, DOTATATE PET/MRI is unlikely to be required to improve target delineation or reduce interobserver variability in cases where good-quality MRI is already highly effective. Variability in GTV delineation between observers may be impacted by meningioma location and lack of consensus on how best to incorporate PET/MRI data for GTV delineation. Larger studies investigating whether the addition of [68]-Ga DOTATATE PET-MRI has an impact of clinical outcomes and toxicity are required, as well as investigations into the cost-benefit of this additional modality.

## Conclusion

For patients with meningiomas due to receive GKSRS, the addition of PET/MRI to MRI for volume delineation resulted in a larger GTV size and significant changes when compared to the MRI-based contour of the treating clinician. However, there was no significant change in interobserver contouring reproducibility as measured by comparison to an objective consensus volume. These findings suggest that caution should be exercised when utilizing PET/MRI imaging for target volume delineation in unselect patients with meningioma for GKSRS, and further studies are needed to define its clinical utility in this setting.

## Supplementary Material

tqad026_Supplementary_Data
