# Supplementary material for: Utility of 68Ga-DOTATATE PET-MRI for Gamma Knife® stereotactic radiosurgery treatment planning for meningioma
Source: Br J Radiol. 2023 Dec 12;97(1153):180–5. doi: 10.1093/bjr/tqad026 (PMC11027229; doi:10.1093/bjr/tqad026)
Supplement: tqad026_Supplementary_Data [file tqad026_supplementary_data.zip › Supplementary Materials.docx]

# **Supplementary Materials**

**Table S1** Summary of DOTATE PET/MRI parameters including the Metabolic Tumour Volume at 50% of the maximum intensity (MTV50), and standardized uptake values (SUV) including minimum (SUV_min_), mean (SUV_mean_), and maximum (SUV_max_). Gross Tumour Volume (GTV) data for treating clinicians GTV_MRI_ contour (Reference) as well as the average GTV_MRI_ (GTV-MRI) and GTV_PET/MRI_ (GTV-PET/MRI) contours.

| **Patient** | **MTV50 (cm3)** | **GTV** | **SUV** | | |
| --- | --- | --- | --- | --- | --- |
|  |  |  | **Min** | **Mean** | **Max** |
| 1 | 0.9 | GTV-Reference | 1.52 | 4.89 | 9.88 |
|  |  | GTV-MRI | 2.10 | 5.43 |  |
|  |  | GTV-PET/MRI | 1.41 | 4.93 |  |
| 2 | 1.5 | GTV-Reference | 5.83 | 24.07 | 43.99 |
|  |  | GTV-MRI | 5.28 | 23.15 |  |
|  |  | GTV-PET/MRI | 3.99 | 20.95 |  |
| 3 | 0.9 | GTV-Reference | 0.36 | 2.70 | 6.95 |
|  |  | GTV-MRI | 0.71 | 3.93 |  |
|  |  | GTV-PET/MRI | 0.87 | 3.86 |  |
| 4 | 0.9 | GTV-Reference | 0.16 | 4.16 | 19.19 |
|  |  | GTV-MRI | 0.67 | 7.02 |  |
|  |  | GTV-PET/MRI | 1.33 | 7.03 |  |
| 5 | 1.5 | GTV-Reference | 0.69 | 8.52 | 21.62 |
|  |  | GTV-MRI | 1.27 | 10.30 |  |
|  |  | GTV-PET/MRI | 1.91 | 9.56 |  |
| 6 | 1.1 | GTV-Reference | 3.00 | 7.26 | 13.42 |
|  |  | GTV-MRI | 1.85 | 7.21 |  |
|  |  | GTV-PET/MRI | 2.57 | 6.97 |  |
| 7 | 1.4 | GTV-Reference | 0.10 | 1.10 | 3.38 |
|  |  | GTV-MRI | 0.14 | 1.31 |  |
|  |  | GTV-PET/MRI | 0.22 | 1.48 |  |
| 8 | 3.6 | GTV-Reference | 0.31 | 2.30 | 3.67 |
|  |  | GTV-MRI | 0.51 | 2.15 |  |
|  |  | GTV-PET/MRI | 0.64 | 2.30 |  |
| 9 | 2.2 | GTV-Reference | 0.33 | 4.24 | 10.63 |
|  |  | GTV-MRI | 1.29 | 4.67 |  |
|  |  | GTV-PET/MRI | 1.28 | 5.88 |  |
| 10 | 1.8 | GTV-Reference | 0.37 | 2.71 | 5.69 |
|  |  | GTV-MRI | 0.39 | 2.77 |  |
|  |  | GTV-PET/MRI | 0.54 | 2.70 |  |
| 11 | 2.6 | GTV-Reference | 0.48 | 2.71 | 5.24 |
|  |  | GTV-MRI | 0.61 | 2.62 |  |
|  |  | GTV-PET/MRI | 0.64 | 2.62 |  |
| 12 | 2.8 | GTV-Reference | 0.35 | 1.92 | 3.52 |
|  |  | GTV-MRI | 0.30 | 1.80 |  |
|  |  | GTV-PET/MRI | 0.50 | 1.93 |  |
| 13 | 1.8 | GTV-Reference | 1.07 | 7.22 | 18.38 |
|  |  | GTV-MRI | 1.00 | 7.14 |  |
|  |  | GTV-PET/MRI | 1.70 | 7.56 |  |
| 14 | 5.3 | GTV-Reference | 1.56 | 6.37 | 10.05 |
|  |  | GTV-MRI | 1.40 | 6.35 |  |
|  |  | GTV-PET/MRI | 1.18 | 6.00 |  |
| 15 | 3.3 | GTV-Reference | 0.77 | 11.14 | 23.21 |
|  |  | GTV-MRI | 0.87 | 12.19 |  |
|  |  | GTV-PET/MRI | 0.79 | 10.99 |  |
| 16 | 0.7 | GTV-Reference | 1.04 | 3.96 | 7.80 |
|  |  | GTV-MRI | 1.23 | 3.80 |  |
|  |  | GTV-PET/MRI | 1.50 | 4.05 |  |
| 17 | 0.6 | GTV-Reference | 2.73 | 21.36 | 46.48 |
|  |  | GTV-MRI | 1.81 | 20.24 |  |
|  |  | GTV-PET/MRI | 2.10 | 20.26 |  |
| 18 | 5 | GTV-Reference | 1.27 | 5.96 | 10.24 |
|  |  | GTV-MRI | 1.12 | 6.26 |  |
|  |  | GTV-PET/MRI | 1.07 | 6.16 |  |
| 19 | 2.7 | GTV-Reference | 0.76 | 3.12 | 8.06 |
|  |  | GTV-MRI | 1.39 | 4.09 |  |
|  |  | GTV-PET/MRI | 1.25 | 3.67 |  |

**Calculation method for windowing levels for GammaPlan**

All information needed to calculate pixel values corresponding to a particular SUV are contained in the DICOM header of the reconstructed PET image data set.

| Descriptor | DICOM tag number |
| --- | --- |
| Radionuclide Total Dose | (0018,1074) |
| Radiopharmaceutical Start Time | (0018,1072) |
| Acquisition Time | (0008,0032) |
| Patient's Weight | (0010,1030) |
| Radionuclide Half Life | (0018,1075) |
| Rescale Slope | (0028,1053) |

where,

= Radionuclide Total Dose (0018,1074)

W(g) = Patient's Weight (0010,1030)

Ta = Acquisition time (0008,0032)

T0 = Radiopharmaceutical Start Time (0018,1072)

T1/2 = Radionuclide Half Life (0028,1053)

Rescale slope = Rescale slope (0028,1053)

*both T1/2 and (Ta-T0) are converted to minutes

A calculator was developed in MS excel so that the required data could be input and the desired window levels were calculated for each patient.

The calculator was developed by a registered nuclear medicine physicist, validated by a second registered nuclear medicine physicist and the windowing was visually approved by the Nuclear Medicine Physician for the first 5 patients.
